# COVID-19's Influence on Ocular Emergency Visits at a Tertiary Referral Center and Its Relationship to Emergency Indications by the American Academy of Ophthalmology

**DOI:** 10.1155/2021/6682646

**Published:** 2021-05-25

**Authors:** Sharon Armarnik, Michael Kinori, Amir Abd Elkader, Sharon Blum Meirovitch, Noa Kapelushnik, Shiran Madgar, Hila Goldberg, Oded Sagiv, Tamara Wygnanski-Jaffe, Ayelet Priel

**Affiliations:** The Goldschleger Eye Institute, Sheba Medical Center, Tel Hashomer, Sackler Faculty of Medicine, Tel Aviv University, Tel Aviv, Israel

## Abstract

**Purpose:**

To examine the trends of ocular emergency admissions during the COVID-19 pandemic at a tertiary care center.

**Methods:**

The study's sample consisted of all patients who were seen in the ophthalmic emergency room (OER) between March 15 and April 15, 2020 (during the COVID-19 pandemic) and in the same time frame of the previous year. The cases were categorized as urgent vs. nonurgent according to the AAO urgency guidelines during the coronavirus period, and the ability to treat the case with telemedicine was evaluated retrospectively.

**Results:**

A total of 553 patients were admitted to the OER during the pandemic period, whereas in the same time frame of the previous year, 1,069 were admitted. The female/male proportion was 526/543 (49.2%/50.8%) in 2019 and 258/259 (46.7%/53.3%) the following year. Age (years, average ± SD) was 44.7 ± 24.5 in 2019 and 47.9 ± 23.4 in 2020. There were more self-referrals in 2020 compared to 2019 (41.1% vs. 32.6%; *p*=0.0004). The time spent in the OER was reduced from 109 ± 74 (minutes, average ± SDV) in 2019 to 73 ± 51 in 2020 (*p* < 0.0001). The most common cause of OER examinations in 2020 was related to the posterior segment of the eye (23.9%), whereas infection and inflammation of the anterior segment were the most common causes in 2019 (35.5%). Urgency by AAO standards was in agreement in 26.7% admissions in 2019 and 35.6% in 2020 (*p*=0.0002).

**Conclusion:**

The COVID-19 pandemic has influenced several aspects of the OER, including the number of referrals, type of ophthalmic emergency, the time spent in the OER, and the need for emergent treatment. Our change in the treatment algorithm was in agreement with the AAO recommendations during the pandemic.

## 1. Introduction

The World Health Organization (WHO) declared coronavirus disease 2019 (COVID-19) as a public health emergency of international concern on January 30, 2020, and as a pandemic on March 11, 2020 [1, 2]. Dr. Li Wenliang, an ophthalmologist from Wuhan, China, was the first physician to raise concern about the coronavirus, and he eventually died from it [3].

In Israel, the first COVID-19 case was confirmed on February 21, 2020—an Israeli citizen who had returned from quarantine on the Diamond Princess ship in Japan. On March 9, the Israeli Ministry of Health (MOH) instructed all Israeli citizens returning from any location overseas to undergo a 14-day home quarantine and, on March 13, published a list of guidelines and restrictions regarding personal movements and social distancing to limit the spread of the infection. Gatherings were restricted, and people were advised to maintain a distance of 2 m (6 ft 7 in) between each other. On March 19, the Prime Minister of Israel declared a national state of emergency, stating that the existing restrictions would be legally enforceable and that violators would be fined. During this period, all cultural establishments and other attractions, as well as coffee shops and restaurants, were closed. However, essential services, including food stores, pharmacies, and banks, remained open. Israelis were not allowed to leave their homes unless absolutely necessary. Some geographic areas (mainly ultraorthodox neighborhoods in Jerusalem and Bnei Brak), where the numbers of confirmed cases spiked, were defined as “restricted zone,” with limited entry and exit. Restrictions on movement were further tightened on March 25 and April 1, when all individuals, except for small children, were instructed to cover their noses and mouths with masks when outdoors. In addition, total quarantine was imposed for a few days.

During the period of major restrictions, as mentioned, from March 15 to April 15, 2020, there was a decrease in the number of visits and a change in the types of ophthalmic emergency room (OER) complaints, as mentioned in different reports from around the world [4, 5]. In addition, a delay in diagnosis and treatment has also been noticed due to patients' fear of exposure to the virus when in clinics and hospitals. On national television, as in other countries [5], health authorities encouraged citizens not to hesitate to seek medical help, especially for health conditions whose risks may increase with delayed diagnosis, for example, heart and vascular diseases.

To learn about the trends during a health threat period, we compared all OER patients that were admitted during the peak of COVID-19 to those during the same period in the previous year. This was an appropriate time to observe the population's behavior under reduced physician care and guidance condition.

## 2. Methods

### 2.1. Study Design and Setting

This study was approved by the local ethics committee of Sheba Medical Center. We conducted a retrospective chart review of patients who were admitted to the OER in our tertiary referral university-affiliated medical center between March 15 and April 15, 2020 (during the period of the COVID-19 quarantine in Israel) compared to the same period in the previous year (March 15 to April 15, 2019).

The information that was collected from each admission included the demographic details such as age, sex, town of origin, ocular history, main complaint, duration of symptoms, and the source of the referral. Information regarding the visit that was collected included the duration of the visit, ophthalmic exam findings, if pupillary dilatation was done, final diagnosis, treatment (if urgent procedure or admission was needed), the recommendations for follow-ups, and their location. Post-factum, cases were classified as “urgent” or “nonurgent” in accordance with the AAO recommendations during the COVID-19 pandemic [6]. We retrospectively assessed the option of using telemedicine in each case. A senior staff from our department reviewed the charts retrospectively for the possibility of resolving the cases only by telemedicine. Each case was assessed as fit or not for telemedicine.

### 2.2. Statistical Analysis

Statistical analysis was carried out using Microsoft Excel 2013 (Microsoft Corporation, Redmond, WA, USA) and GraphPad Prism version 6.0 for Windows (GraphPad Software, San Diego, CA, USA). Chi-square test was used to evaluate the statistical significance of the difference in proportions between study groups, and a *t*-test was used to evaluate the statistical significance of the difference in the quantitative parameters between the study groups. A *p* value of 0.05 or less was considered statistically significant.

## 3. Results

### 3.1. Demographic Variables

A total of 1,622 patients—1,069 in 2019 and 553 in 2020 (48.27% reduction)—were examined in the OER during the periods studied.  Table 1 summarizes the demographics of the study population for each year.

During the weekend (Friday and Saturday) in 2019, 283 (26.4%) patients were examined, whereas in 2020, only 89 (16.1%) subjects were examined during the weekend (*p* < 0.0001). Of the 1,622 patients, 348 (32.6%) and 229 (41.4%) were self-referred in 2019 and 2020 (*p*=0.0004), respectively.

### 3.2. Diagnoses, Management, and Outcome

**Figure 1:**
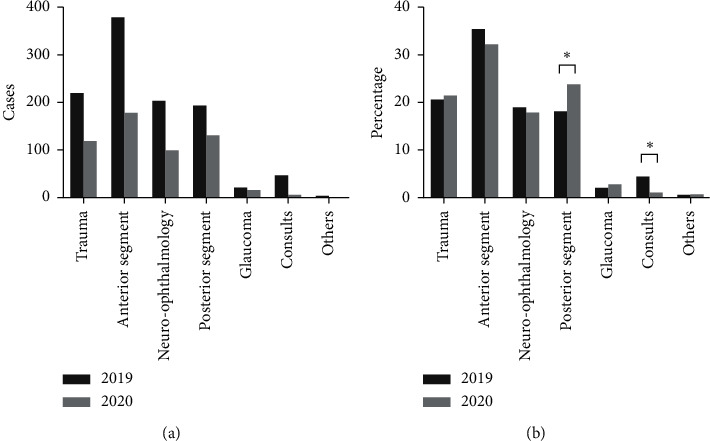
The distribution of patients grouped by diagnosis in the OER during the COVID-19 pandemic (2020) and in the previous year (2019). (a) The number of patients. (b) The percentage of patients.

During examinations, pupillary dilation was carried out on 867 (81.1%) patients in 2019 and 412 (74.5%) in 2020 (*p*=0.002). The time spent in the OER was 109 ± 74 (minutes, average ± SD) in 2019, which decreased to 73 ± 51 (minutes, average ± SD) in the following year (*p* < 0.0001). Hospitalization was needed in 56 (5.2%) patients during the period in 2019 and 39 (7.1%) patients in the following year (*p*=0.14). Emergency procedures were carried out in 147 (13.8%) patients in 2019 and 117 (21.2%) in 2020 (*p*=0.0001). These procedures are summarized in Table 2. We have subdivided the diagnosis into seven groups according to the main diagnosis: trauma, anterior segment, neuro-ophthalmology, posterior segment, glaucoma, consults, and others. Others included orbital tumors, prolapse of orbital fat, and prosthesis issues. Their distribution is shown in Figures 1(a) and 1(b).

A follow-up in the Ophthalmology Department of Sheba Medical Center was recommended for 369 (34.5%) cases in 2019 and 218 (39.4%) cases in 2020 (*p*=0.051).

Of the 23 (4.16%) patients in 2020 who underwent a COVID-19 test (PCR test), only two tested positive.

### 3.3. Treatment Delay, Justification on Arrival, AAO Urgent vs. Nonurgent Visits, and Telemedicine

A delayed admission was defined as a sign or symptom that had commended days before the patient sought medical help. We found 23 (2.2%) OER admissions in 2019 and 14 (2.5%) in 2020 that were considered delayed admissions. Some of the reasons for the delay during 2020 were isolation and fear of leaving the house. Justification for admission to the emergency room for treatment, evaluated by the reviewer, was noted in 535 (50%) cases in 2019 and 339 (61.3%) cases in 2020 (*p* < 0.0001). Urgency by AAO standards [6] was in agreement in 285 (26.7%) cases in 2019 and 197 (35.6%) admissions in 2020 (*p*=0.0002). A total of 169 (15.8%) in 2019 and 90 (16.3%) in 2020 of all the cases treated in the OER were assumed to be resolved using telemedicine alone as a tool in the emergency room.

## 4. Discussion

COVID-19 was declared a pandemic on March 11, 2020, by the WHO, and two days later, the Israeli Ministry of Health (MOH) [7] published a list of guidelines and restrictions regarding personal movements and social distancing. As of April 29, 2020, a total of 2,995,758 laboratory-confirmed cases had been documented globally [8]. On the same day, there were a total of 215 deaths, 115 patients with serious illness, and 15,834 confirmed infected patients in Israel [7].

We found a 51.73% reduction in patients' exams in the OER during the pandemic, compared with the same time frame the previous year as was shown by Wickham et al. [5]. A greater decrease, reaching 74%, was observed by Posarelli at el. during the lockdown period [4]. This could be explained by governmental restrictions and fear of encountering the coronavirus during the time of quarantine.

Pediatric admissions to the OER, as shown in Table 1, have also decreased significantly. This reduction might be explained by the less exposure to contagious illnesses and trauma during the quarantine period, especially amongst the pediatric age group. Since the age group with the highest risk for infection is 65 and older, as stated by the Centers for Disease Control and Prevention [9], it is reasonable to assume that the average age at presentation would decline. However, the percentage of OER admissions by the older age group did not decline, as shown in Table 1, and was shown in previous reports [4]. On the contrary, the average age at presentation was even higher (44.7 ± 24.5 in 2019 and 47.9 ± 23.4 in 2020 (*p*=0.01)). This could be explained by the repeated announcements by the Israeli MOH to refrain from postponing urgent care for health issues not related to the coronavirus.

Fewer patients visited the OER on weekends during the pandemic (26.4% compared with 16.1%, *p* < 0.0001). This could result from closed businesses on weekdays, allowing working-aged people to seek medical help on those days. Moreover, ophthalmology outpatient clinics and private practices were usually closed, prompting patients to go to the OER instead. This explains the increased number of self-referrals during the COVID-19 period.

As guidance, our ophthalmology team was instructed to give priority to the main complaint of the OER patients and to minimize unnecessary conversations and interactions in order to limit the amount of time the patients spent in the hospital and to protect the medical staff. Minimized interaction was shown in our study through two aspects: (1) the time spent in the OER decreased by 33% (from an average of 109 minutes in 2019 to 73 minutes in 2020 (*p* < 0.0001)) and (2) the pupillary dilation rates were reduced in 2020, compared with 2019. Wickham et al. found a similar trend with a reduction of average wait time to 1 h from registration to discharge [5].

We classified seven groups of clinical diagnoses: posterior segment, anterior segment, trauma, neuro-ophthalmology, glaucoma, inpatient consults, and others. The most common cause of OER examinations in 2020 was related to the posterior segment of the eye (23.9%), whereas infection and inflammation of the anterior segment were the most common causes in 2019 (35.5%). An increase in the percentage of posterior segment cases was observed as well by Posarelli and coworkers [4]. A reduction in the number of cases was seen in all the groups during the pandemic. The decrease in ocular traumas [4, 10] and infectious conjunctivitis [10] had been pointed and explained by social distancing. The reduction in other categories, which are sight threatening, was hypothesized to occur by the patients' reluctance to be exposed to the virus in hospitals. In our cohort, there was no significant difference in the distribution of cases within the groups between 2019 and 2020, except for inpatient consults and posterior segment cases. The inpatient consults had decreased in 2020; the obvious explanation is the desire to minimize unnecessary inpatient traffic. This reduction was observed even more during the lockdown period compared to prelockdown during the pandemic [4].

Admission was needed in 56 (5.2%) patients during 2019 and in 39 (7.1%) patients in the following year. The government's movement restriction may have influenced the increase in the percentage of cases favoring hospitalization over outpatient treatment. The same limitation may also have contributed to the trend of an increase in the percentage of follow-ups in the Ophthalmology Department of Sheba Medical Center, from 34.5% (369 cases) in 2019 to 39.4% (218 cases) in 2020 (*p*=0.051).

The AAO has published a list of ophthalmic diagnoses that are considered as urgent to help clinicians balance the fear of disease transmission and the need to give timely medical care [6]. Lou et al. even published a workflow for COVID-19 screening and the management of patients with eye emergencies [11]. Dr. Leung and her colleagues published a summary of all published guidelines, including the AAO, from ophthalmic organizations that provided clarification of emergent, urgent, and elective ocular conditions during the COVID-19 pandemic [12]. They emphasized the need to learn from the experience of one heavily impacted population and use it to mitigate the effects in other countries. We graded our cases based on two separate measures: (1) an assessment by the reviewer of the urgency of a condition as a “justification” to arrive to the OER and (2) categorization of the urgent or nonurgent cases according to the AAO criteria. Our findings show that 61.3% of the diagnoses in 2020 and 50% in 2019 (*p* < 0.0001) were justified as urgent, as was seen by the Posarelli group [4], reflecting the natural selection of patients under quarantine. Based on the AAO criteria, the same trend was observed because 35.6% of patients in 2020 were considered “urgent,” whereas only 26.7% were considered so in 2019 (*p* < 0.0002). Emergency procedures were carried out in 147 (13.8%) and 117 (21.2%) patients in 2019 and 2020 (*p*=0.0001), respectively, reflecting the increase of more severe cases in 2020.

To better control and manage the rapid spread of COVID-19, both developed and developing countries can improve the efficiency of their health system by replacing a proportion of face-to-face clinical encounters with telehealth. Recent technological advancement facilitates this reform, but there is a need for national or statewide rules and regulations to be adapted accordingly [13]. Telemedicine or telehealth can play an important role in certain specialties. Langer and Bernardini described the benefits of using telemedicine during the pandemic as a tool to evaluate oculofacial patients and postoperatively manage the patients in selected cases [14]. Moorfields Eye Hospital has launched digital consultations after the beginning of the lockdown; they were shown to be effective for minor symptoms [5]. In our postfactum assessment, we found that only 16% (15.8% in 2019 and 16.3% in 2020) of all cases could have been treated via telemedicine and could have avoided in-person admissions to the OER. Even though telemedicine can become a great way to resolve problems without the risk of infection, it does not fit most OER cases since many of the diagnoses are more subtle than meets the eye. The percentage of patients who would have gained from telemedicine is not high, 169 (15.8%) in 2019 and 90 (16.3%) in 2020, and even if an emergency treatment can be given through it, a follow-up visit would be needed for most if not all patients. Telemedicine could help to relieve a busy OER in times of a pandemic in certain diagnoses (e.g., eyelid pathologies and conjunctivitis) and could be considered as an option when all liability issues are settled.

At the beginning of the pandemic, our department and clinic followed a strict approach to protect the staff and ensure the health of patients. We have treated a few patients that tested positive for COVID-19; some were found to be positive retrospectively. However, not a single staff member was infected. Nevertheless, we must continue to take extra precautions because ophthalmologists are continuing to treat patients during the pandemic and during the upcoming second wave of COVID-19.

It is still unclear how many patients have suffered or are suffering from vision loss or ophthalmic complications as a result of treatment delay during the pandemic as been shown already [5]. This information is crucial, and unfortunately, it will be revealed only in the future, and we should plan for recovery strategies from the hidden burden.

## 5. Conclusion

COVID-19 pandemic has changed the OER in several aspects, mainly seen as a reduction of almost 50% in all visits. Posterior segment was the only subspecialty that the percentage out of all visits increased. The main reduction was seen in the number of referrals, almost 90% reduction. The changes are related to the quarantine limitations, on the one hand, and to the natural selection of urgent cases, on the other hand. These changes should be considered for creating guidelines such as the ones proposed by the AAO in order to prioritize urgent diagnosis.

## Figures and Tables

**Table 1:** The demographics of the study population in each year.

	2019 (*n* = 1069)	2020 (*n* = 553)	*p* value
Female/male (%)	526/543 (49.2%/50.8)	258/259 (46.7%/53.3)	ns
Age (years, average ± SD)	44.7 ± 24.5	47.9 ± 23.4	0.01
Children (<18 years)	192 (18.0%)	69 (12.5%)	0.004
Endemic origin	202 (18.9%)	89 (16.1%)	ns

^∗^ns: nonsignificant.

**Table 2:** Characteristics of the OER admissions and the outcome of each admission.

	2019 (*n* = 1069)	2020 (*n* = 553)	*p* value
Pupillary dilation in the ER	867 (81.1%)	412 (74.5%)	0.002
Time in the ER (minutes, average ± SD)	109 ± 74	73 ± 51	<0.0001
Hospitalization needed	56 (5.2%)	39 (7.1%)	ns
Emergency procedure needed	147 (13.8%)	117 (21.2%)	0.0001

^∗^ns: nonsignificant. ER: emergency room.

## Data Availability

The data used to support the findings of this study will be made available from the corresponding author upon request; the main Excel table is found with the corresponding author.
